# Atypical Chikungunya Virus Infections in Immunocompromised Patients

**DOI:** 10.3201/eid1606.091115

**Published:** 2010-06

**Authors:** Adrian C.L. Kee, Samantha Yang, Paul Tambyah

**Affiliations:** National University Hospital, Singapore

**Keywords:** Chikungunya virus, immunocompromised, viruses, arbovirus, letter, *Suggested citation for this article*: Kee ACL, Yang S, Tambyah P. Atypical chikungunya virus infections in immunocompromised patients. Emerg Infect Dis [serial on the Internet]. 2010 Jun [date cited]. Available from http://www.cdc.gov/EID/content/16/6/1038.htm

**To the Editor:** Chikungunya fever was first described in Tanganyika (now Tanzania) in 1952 and is now emerging in Southeast Asia. Chikungunya virus (CHIKV) infection, a self-limiting febrile illness, shares similarities with dengue fever such as headache and myalgia. Additionally, patients with CHIKV infection typically have arthralgia, arthritis, and tenosynovitis (*1*). Although usually benign, CHIKV infection may on rare occasions lead to neurologic and hepatic manifestations with high illness and mortality rates (*2*). We report 2 immunocompromised patients with CHIKV infection associated with peritonitis, encephalitis, and secondary bacterial infections.

Patient A, a 66-year-old Singaporean-Chinese man, had a history of chronic renal disease secondary to obstructive uropathy. His baseline creatinine level was 300–400 μmol/L. For 3 years, he had ingested traditional Chinese medicine, which we suspect was contaminated by steroids because he appeared cushingoid. An outbreak of CHIKV infection was reported at his workplace. He was admitted to National University Hospital, Singapore, in July 2008 with abdominal pain, vomiting, and fever of 1 day. He had no joint symptoms. Clinically, he had systemic inflammatory response syndrome complicated by acute-on-chronic renal failure. His creatinine level was elevated at 921 μmol/L on admission. A complete blood count showed leukocytosis (19.24 × 10^9^ cells/L) with neutrophilia and thrombocytopenia (62 × 10^9^ cells/L). Initial blood and urine cultures and serologic results were negative for dengue virus, but serum reverse transcription–PCR (RT-PCR) and indirect immunofluorescent assay for immunoglobulin G (IgG) (Euroimmun Medizinische Labordiagnostika, Lubeck, Germany) and IgM (CTK Biotech, Inc, San Diego, CA, USA) were positive for CHIKV (*3*,*4*). Computed tomographic scans of the abdomen showed dilated small bowel loops.

An urgent laparotomy did not show bowel perforation, but peritoneal cultures yielded *Klebsiella pneumoniae*, *Escherichia coli,* and *Candida glabrata*, and RT-PCR from the concentrated peritoneal fluid was positive for CHIKV (*3*). He was administered appropriate antimicrobial drugs. He required repeat laparotomies because of elevated intraabdominal pressure. He subsequently received broad spectrum antimicrobial drugs to treat secondary intraabdominal infections caused by *P. aeruginosa* and *Enterococcus faecalis*.

Ventilator-associated pneumonia also developed. Despite maximal support and prolonged antimicrobial therapy, this patient died after 5 months of hospitalization.

Patient B, a 45-year-old Malaysian–Chinese man with diabetes mellitus, had undergone a cadaveric liver transplant in 2001 for hepatitis B liver cirrhosis. He was receiving immunosuppressants (azathioprine and prednisolone). He was admitted in August 2008 after experiencing fever, headache, and abdominal bloating for 3 days. He had no neurologic symptoms. Acute self-limiting febrile illnesses with arthritis had occurred in his hometown; CHIKV infections were suspected.

Results of his examination on admission were normal, except for bilateral enlarged cervical lymph nodes. Chest radiograph results were unremarkable. He had mild transaminitis (alanine aminotransferase 173 U/L, aspartate aminotransferase 170 U/L), elevated C-reactive protein (107 mg/L), and thrombocytopenia (120 × 10^9^ cells/L) without leukocytosis. Results of comprehensive serum and urine microbial studies were negative for posttransplant infections. Results of serum RT-PCR were negative for CHIKV, but IgG and IgM tests were positive for CHIKV.

Brain magnetic resonance imaging was performed because of the patient’s persistent severe headache and transient drowsiness. It showed several nonspecific areas of enhancement, which suggested encephalitis, given the clinical scenario (Figure). However, a lumbar puncture was not performed, and hence, whether the patient’s cerebrospinal fluid contained CHIKV could not be determined. Bilateral frontoparietal white matter lesions with restricted diffusion has been suggested as an early sign of viral encephalitis (*5*). However, a retrospective series demonstrated that, in CHIKV encephalitis, abnormalities on magnetic resonance imaging were uncommon, and no pathognomonic features were found (*6*).

**Figure:**
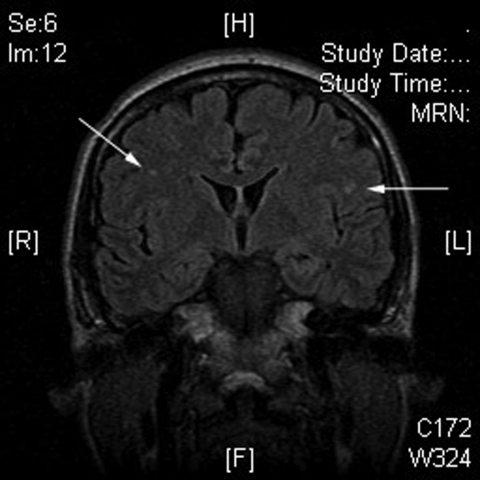
Magnetic resonance imaging of the brain of patient B, showing several nonspecific areas of enhancement (arrows), which suggests encephalitis, given the clinical scenario.

Hospital-acquired pneumonia also developed and was treated with broad-spectrum antimicrobial drugs. Bronchoscopic cultures were negative for CHIKV. The patient responded well to antimicrobial drugs, and his mental status was normal on discharge. He possibly had encephalitis associated with CHIKV infection, complicated by secondary hospital acquired pneumonia.

In this case, CHIKV was detected in peritoneal fluid, but because of the positive bacterial cultures, we are not confident about its causative role in patient A’s peritonitis. Although a series reported that 6 patients with CHIKV infection had perforated jejunal diverticula while receiving long-term nonsteroidal antiinflammatory drugs and steroids (*7*), the perforations were likely secondary to prolonged steroid use rather than CHIKV infection. In addition, both immunocompromised patients in our study had their CHIKV infections secondarily complicated by nosocomial infections. We note that other viral infections have been associated with bacterial translocation and secondary nosocomial infections (*8*). Whether these infections were linked to CHIKV infection or to the underlying chronic immunosuppressed state is unclear.

Both of our patients did not have the joint manifestations that are characteristic of CHIKV infection (*9*). More prospective studies are required to determine the full spectrum of clinical features of CHIKV infection in immunocompromised patients. Recently identified biomarkers may predict patients at risk for complications but we were unable to study them in our patients (*10*). Although most cases of CHIKV infection are self-limiting, clinicians should be alert to atypical presentations and severe complications in immunosuppressed patients.
